# The Effect of Intraventricular Hemorrhage on Brain Development in Premature Infants: A Synthetic MRI Study

**DOI:** 10.3389/fneur.2021.721312

**Published:** 2021-09-09

**Authors:** Chunxiang Zhang, Xin Zhao, Meiying Cheng, Kaiyu Wang, Xiaoan Zhang

**Affiliations:** ^1^Department of Radiology, The Third Affiliated Hospital of Zhengzhou University, Zhengzhou, China; ^2^Institute of Neuroscience, Zhengzhou University, Zhengzhou, China; ^3^GE Healthcare, MR Research China, Beijing, China

**Keywords:** synthetic MRI, intraventricular hemorrhage, premature infants, stroke, brain injury

## Abstract

**Objectives:** Synthetic MRI can obtain multiple parameters in one scan, including T1 and T2 relaxation time, proton density (PD), brain volume, etc. This study aimed to investigate the parameter values T1 and T2 relaxation time, PD, and volume characteristics of intraventricular hemorrhage (IVH) newborn brain, and the ability of synthetic MRI parameters T1 and T2 relaxation time and PD to diagnose IVH.

**Materials and methods:** The study included 50 premature babies scanned with conventional and synthetic MRI. Premature infants were allocated to the case group (*n* = 15) and NON IVH (*n* = 35). The T1, T2, PD values, and brain volume were obtained by synthetic MRI. Then we assessed the impact of IVH on these parameters.

**Results:** In the posterior limbs of the internal capsule (PLIC), genu of the corpus callosum (GCC), central white matter (CWM), frontal white matter (FWM), and cerebellum (each *p* < 0.05), the T1 and T2 relaxation times of the IVH group were significantly prolonged. There were significant differences also in PD. The brain volume in many parts were also significantly reduced, which was best illustrated in gray matter (GM), cerebrospinal fluid and intracranial volume, and brain parenchymal fraction (BPF) (each *p* < 0.001, *t* = −5.232 to 4.596). The differential diagnosis ability of these quantitative values was found to be excellent in PLIC, CWM, and cerebellum (AUC 0.700–0.837, *p* < 0.05).

**Conclusion:** The quantitative parameters of synthetic MRI show well the brain tissue characteristic values and brain volume changes of IVH premature infants. T1 and T2 relaxation times and PD contribute to the diagnosis and evaluation of IVH.

## Introduction

Intraventricular hemorrhage (IVH) is a common complication of premature infants and a typical form of intracranial hemorrhage in premature infants. Despite advances in clinical management, the absolute number of cases is still high due to the increase in the survival rate of premature infants and the detection rate of minor diseases (1). In addition, serious complications may occur, such as hydrocephalus and/or parenchymal hemorrhage after hemorrhage. IVH is closely related to long-term neurodevelopmental disorders (2). Therefore, it is necessary to identify IVH at a very early stage so that they can benefit from early neurological rehabilitation.

The long-term neurological outcome of IVH syndrome is still controversial and is still an active area of research. It was believed that IVH did not increase the risk of neurodevelopmental disorders related to preterm birth (3). Some recent opinions believe that IVH premature infants have dysfunction and immature white matter microstructure. Patients with mild IVH are at an increased risk of neurological impairment. IVH adversely affects the cerebellum growth of premature infants (4, 5). Synthetic MRI is an emerging available technique that combines conventional high-spatial resolution MRI images with quantitative T1, T2 relaxometry, PD, and volumetry.

Synthetic MRI is a quantitative imaging technique that can measure inherent relaxometry of T_1_, T_2_, and proton density (PD). In addition, it can reconstruct multiple image contrasts in one scan, which can save about 13 min of scan time compared with traditional MRI, and it can provide new information. Brain volume segmentation and calculation can also be performed through image post-processing. Synthetic MRI takes less time and obtains more image information. Therefore, this is especially suitable for some acute patients such as stroke. Although this emerging imaging technique has been described in some neurological fields, there has been no report on the use of synthetic MRI in children with IVH.

The objectives of this study were to investigate the trend of local relaxation time and brain volume changes in IVH premature infants, and to study the diagnostic ability of quantitative parameters of synthetic MRI in IVH premature infants.

## Materials and Methods

### Subjects

The study included premature babies scanned from January 2020 to March 2021, and for which both conventional MRI and synthetic MRI were performed. The exclusion criteria included congenital malformations, congenital TORCH infection, periventricular leukomalacia, high-grade IVH, and poor image quality. The case group inclusion criteria were premature infants with low-grade IVH. The inclusion criteria of the control group were premature infants, no intracranial hemorrhage, no infectious diseases, no genetic diseases, etc. They had an MRI scan because they failed the hearing screening. Finally, 15 cases in the IVH group and 35 cases in the NON IVH group were included, and we obtained the informed consent of the parents.

### MRI Protocol

All infants were sedated with dexmedetomidine hydrochloride injection, 0.75–1 μg/kg. Then the babies were placed on the MRI bed, and the ears were plugged with earplugs to protect hearing and reduce motion artifacts. MR scan was carried out on a 3.0 T MR scanner (Pioneer, GE Healthcare, Milwaukee, WI, USA) in a supine position using a 16-channel head coil, with T1WI (TR = 2,198 ms, TE = 4.62 ms), T2WI (TR = 8,000 ms, TE = 6.22 ms), and T2 FLAIR (TR = 2,000 ms, TE = 2.32 ms, b value =0.1000 mm^2^/s). SyMRI software was used to calculate T1 and T2 relaxation maps, and T1WI (TR = 650 ms, TE = 10 ms), T1 FLAIR (TR = 500 ms, TE = 90 ms), T2WI (TR = 4,500 ms, TE = 100 ms), T2 FLAIR (TR = 15,000 ms, TE = 90 ms), and PD (TR = 8,000 ms, TE = 10 ms) images were reconstructed and synthesized for diagnostic evaluation. Their parameters were the same and are as follows: slice thickness: 4.0 mm, field of view: 220 × 186 mm. Scan time was 4 min. Then the professional manually outlined the region of interest (ROI) on the T1 image of SyMRI: posterior limbs of the internal capsule (PLIC), genu of the corpus callosum (GCC), parietal white matter (PWM), frontal white matter (FWM), thalamus (TH), central white matter (CWM), occipital white matter (OWM), and cerebellum. The ROI size was controlled within (10 ± 2) mm^2^. In order to avoid researcher bias, all ROIs were described by the same pediatric neuroradiologist, who had 10 years of experience in neonatal MRI and know nothing about grouping. The average T1 and T2 relaxation values of the selected ROI were automatically provided by the SyMRI software.

Based on the obtained T1, T2, and PD, we also calculated the gray matter (GM) volume, cerebrospinal fluid (CSF) volume, brain parenchymal volume (BPV), and intracranial volume (ICV) of the whole brain on the SyMRI software. The BPV was calculated as the sum of WM, GM, and NoN (tissue with characteristics that deviate from WM, GM, and CSF). ICV was calculated as the sum of BPV and cerebrospinal fluid. The brain parenchymal fraction (BPF) was BPV divided by ICV. Skull stripping, to remove tissue outside the intracranial volume, was automatically performed by an existing module in this software.

### Statistical Analysis

All data were statistically analyzed with SPSS version 21 (IBM Corporation, Armonk, NY, USA). The normality of each variable was tested using the Shapiro–Wilk test. To determine the difference in clinical characteristics between the two groups, we used the Mann–Whitney *U*-test of continuous and chi-square test of categorical variables. The quantitative values (T1, T2, and PD), tissue volume, and tissue score of the two groups in each segmentation area were compared, using the Mann–Whitney *U*-test or Student *t*-test.

In addition, the ROC curve and the area under the curve (AUC) were used to evaluate the ability of relaxation time diagnostic. The AUC between 0.7 and 0.8 was considered acceptable, and 0.8–0.9 was considered excellent. *p* < 0.05 were considered to represent a statistically significant result. Graphs were prepared with GraphPad Prism version 8 (GraphPad Software, San Diego, CA, USA).

## Results

### Study Participants

Fifty premature babies were included in the study, 15 cases showed IVH (9 males, average gestational age 29.38 ± 2.58 weeks), and 35 cases of brain MRI showed normal (15 males, average gestational age 30.45 ± 3.34 weeks). Gestational age (GA) at birth, postmenstrual age (PMA) at scan, birth weight, Apgar score, gender, and delivery method had no statistical significant difference (*p* > 0.05) (as shown in Table 1).

**Table 1:** Demographic characteristics.

	**IVH group(n=15)**	**NON IVH group (*n* = 35)**	***P*-value**
GA(week)	29.38 ± 2.58	30.45 ± 3.34	0.278
Mean PMA at MRI (week)	31.53 ± 3.08	33.15 ± 3.23	0.106
Birth weight (g)	1186.20 ± 260.59	1452.09 ± 860.78	0.249
Birth weight at MRI (g)	2420.11 ± 710.22	2120.71 ± 210.08	0.193
Apgar score (1 min)	7.87 ± 1.19	8.09 ± 1.34	0.589
Apgar score (5 min)	7.80 ± 1.52	7.00 ± 1.53	0.097
male	9 (60%)	15 (42.9%)	0.266

*GA, gestational age; PMA, postmenstrual age. There were no significant differences between these two groups (P > 0.05)*.

### Quantitative Parameters in the IVH and NON IVH Groups

**Figure 1:**
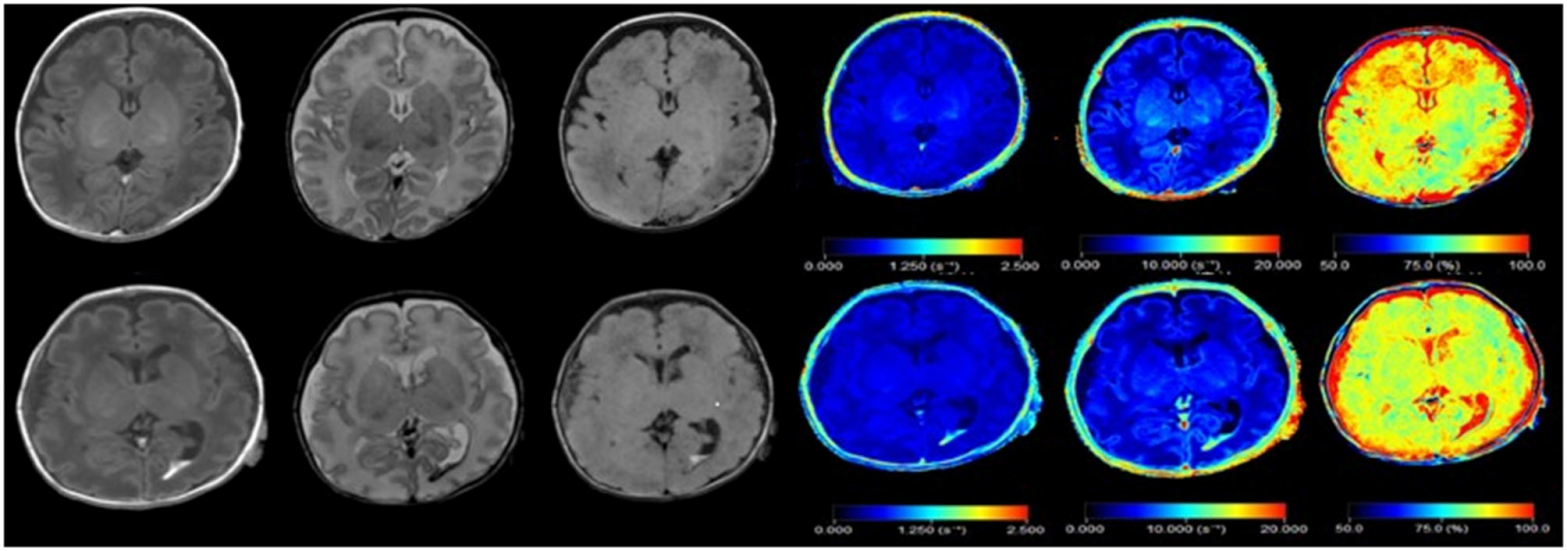
Synthetic images of a normal premature infant, and premature infant with intraventricular hemorrhage. Upper row: Synthetic MRI of normal preterm infants, in order of T1, T2, T2 FLAIR, R1, R2, PD. Bottom row: Synthetic MRI of premature infants with intraventricular hemorrhage, in order of T1, T2, T2 FLAIR, R1, R2, PD. (R1 = 1/T1, R2 = 1/T2).

The T1, T2 relaxation times and PD were used to evaluate the differences between the two groups of premature brain development (Figure 1 and Table 2). In the PLIC, GCC, CWM, FWM, and cerebellum (all *p* < 0.05), the T1 and T2 relaxation times in the IVH group were significantly prolonged. At the same time, the T1 relaxation time in TH was also significantly longer (*p* = 0.003, *t* = 3.159). The significant difference in PD between the two groups was mainly manifested in PLIC (*p* = 0.044, *t* = 2.072), CWM (*p* = 0.003, *t* = 3.171), FWM (*p* = 0.017, *t* = 2.469), and cerebellum (*p* = 0.035, *t* = 2.164).

**Table 2:** Quantitative values in IVH and NON IVH groups.

	**IVH group (*n* = 15)**	**NON IVH group (*n* = 35)**	***P*-value**	***t*-value**
**T1 relaxation**				
PLIC	1575.07 ± 160.73	1316.37 ± 209.75	0.000^*^	4.261
TH	1539.67 ± 204.99	1360.69 ± 174.03	0.003^*^	3.159
GCC	1962.73 ± 251.94	1780.14 ± 172.58	0.005^*^	2.973
CWM	1989.33 ± 531.55	1638.80 ± 245.76	0.026^*^	2.445
FWM	2011.67 ± 186.60	1795.46 ± 174.27	0.000^*^	3.937
PWM	1842.33 ± 264.37	1682.66 ± 285.89	0.071	1.849
OWM	1879.80 ± 289.91	1786.26 ± 219.19	0.216	1.253
Cerebellum	1708.13 ± 220.89	1504.94 ± 140.04	0.000^*^	3.926
**T2 relaxation**				
PLIC	148.27 ± 23.27	130.63 ± 16.43	0.004^*^	3.059
TH	132.40 ± 16.24	126.54 ± 19.14	0.306	1.035
GCC	186.07 ± 32.11	165.69 ± 31.83	0.044^*^	2.069
CWM	216.40 ± 55.90	165.69 ± 26.87	0.004^*^	3.352
FWM	231.93 ± 36.63	193.40 ± 28.06	0.000^*^	4.053
PWM	198.60 ± 40.90	175.03 ± 38.09	0.056	1.962
OWM	206.73 ± 48.34	179.74 ± 28.56	0.059	2.017
Cerebellum	178.67 ± 35.06	147.11 ± 24.62	0.001^*^	3.642
**PD**				
PLIC	83.38 ± 4.90	80.44 ± 4.47	0.044^*^	2.072
TH	82.07 ± 2.18	81.53 ± 2.57	0.480	0.712
GCC	80.72 ± 4.58	80.56 ± 5.47	0.919	0.102
CWM	86.35 ± 3.39	83.46 ± 2.75	0.003^*^	3.171
FWM	85.27 ± 1.13	83.75 ± 3.22	0.017^*^	2.469
PWM	85.40 ± 1.57	84.42 ± 3.22	0.268	1.121
OWM	85.70 ± 2.92	85.97 ± 2.88	0.761	−0.305
Cerebellum	87.36 ± 3.86	84.17 ± 5.12	0.035^*^	2.164

*PLIC, posterior limbs of the internal capsule; GCC, genu of the corpus callosum; PWM, parietal white matter; FWM, frontal white matter; TH, thalamus; CWM, Central white matter; OWM, occipital white matter; WM, white matter*.

* *P < 0.05 is defined as the significance level*.

Although the T1 relaxation time of WM (*p* = 0.071, *t* = 1.849) and OWM (*p* = 0.216, *t* = 1.253) was longer than the NON IVH group, the difference was not significant. T2 relaxation time had no significant difference in TH (*p* = 0.306, *t* = 1.035), PWM (*p* = 0.056, *t* = 1.962), and OWM (*p* = 0.059, *t* = 2.017). The OWM (*p* = 0.761, *t* = −0.305) showed that the PD value of the case group was lower, compared with the control group.

**Figure 2:**
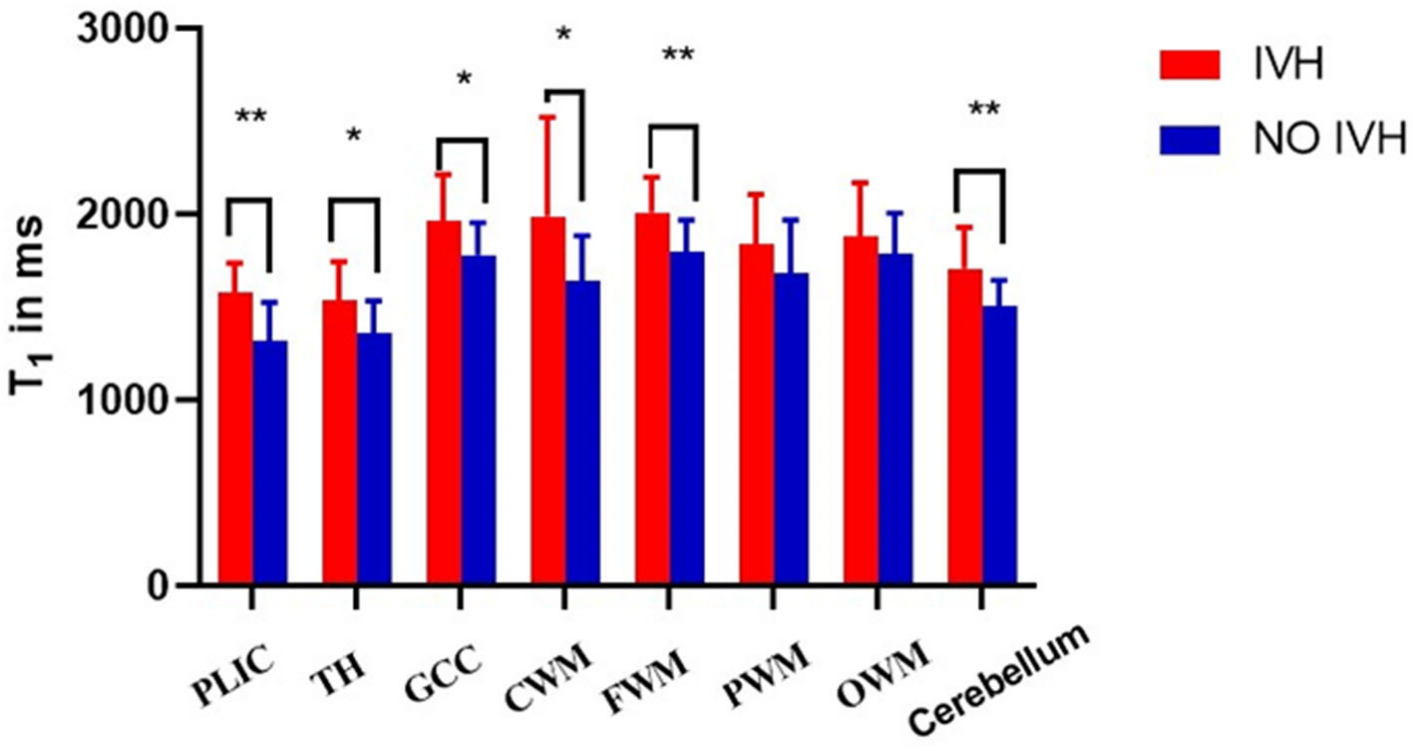
Differences in T1 values between IVH and NON IVH groups. The IVH group is displayed in red and the NON IVH group in blue. PLIC, posterior limbs of the internal capsule; TH, thalamus; GCC, genu of the corpus callosum; CWM, Central white matter; FWM, frontal white matter; PWM, parietal white matter; OWM, occipital white matter. *p < 0.05, **p < 0.001.

**Figure 3:**
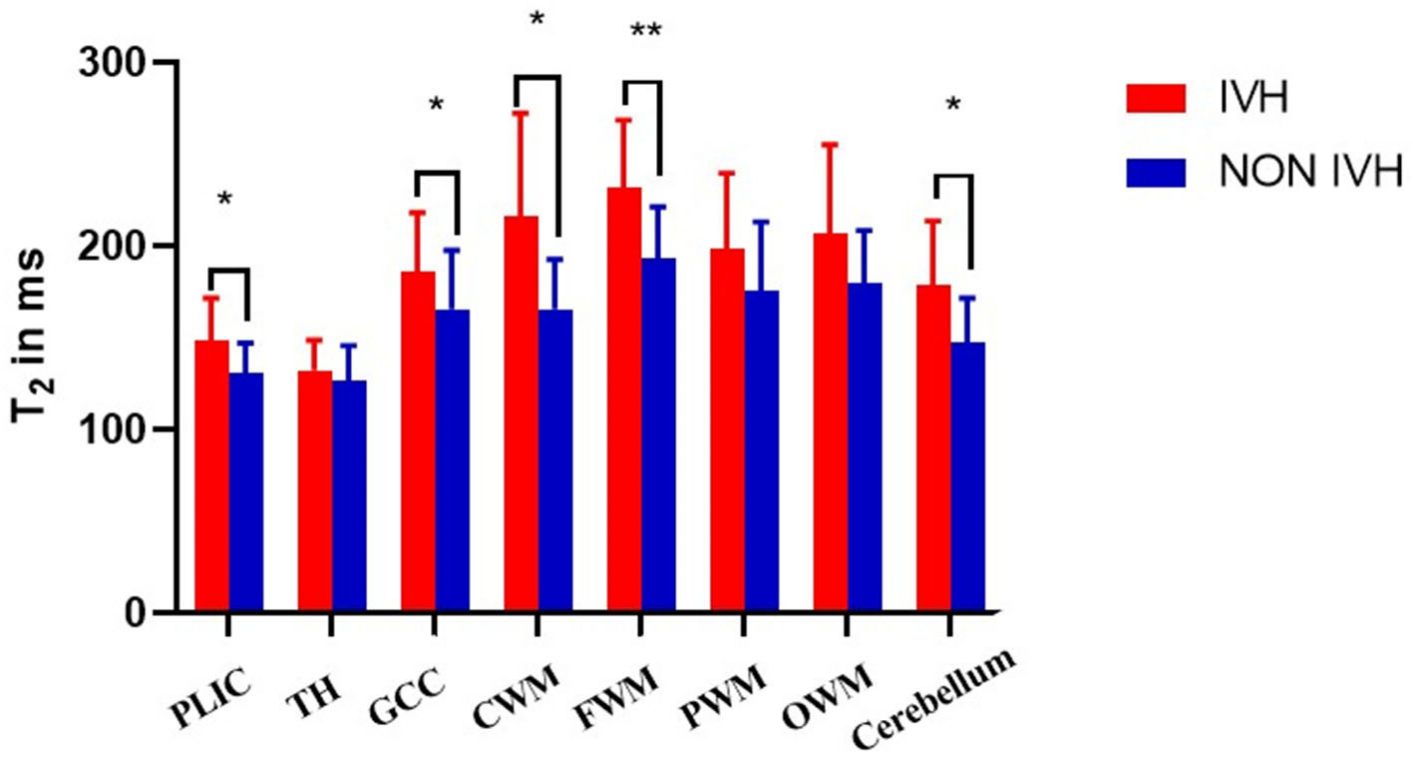
Differences in T2 values between IVH and NON IVH groups. The IVH group is displayed in red and the NON IVH group in blue. PLIC, posterior limbs of the internal capsule; TH, thalamus; GCC, genu of the corpus callosum; CWM, Central white matter; FWM, frontal white matter; PWM, parietal white matter; OWM, occipital white matter. *p < 0.05, **p < 0.001.

**Figure 4:**
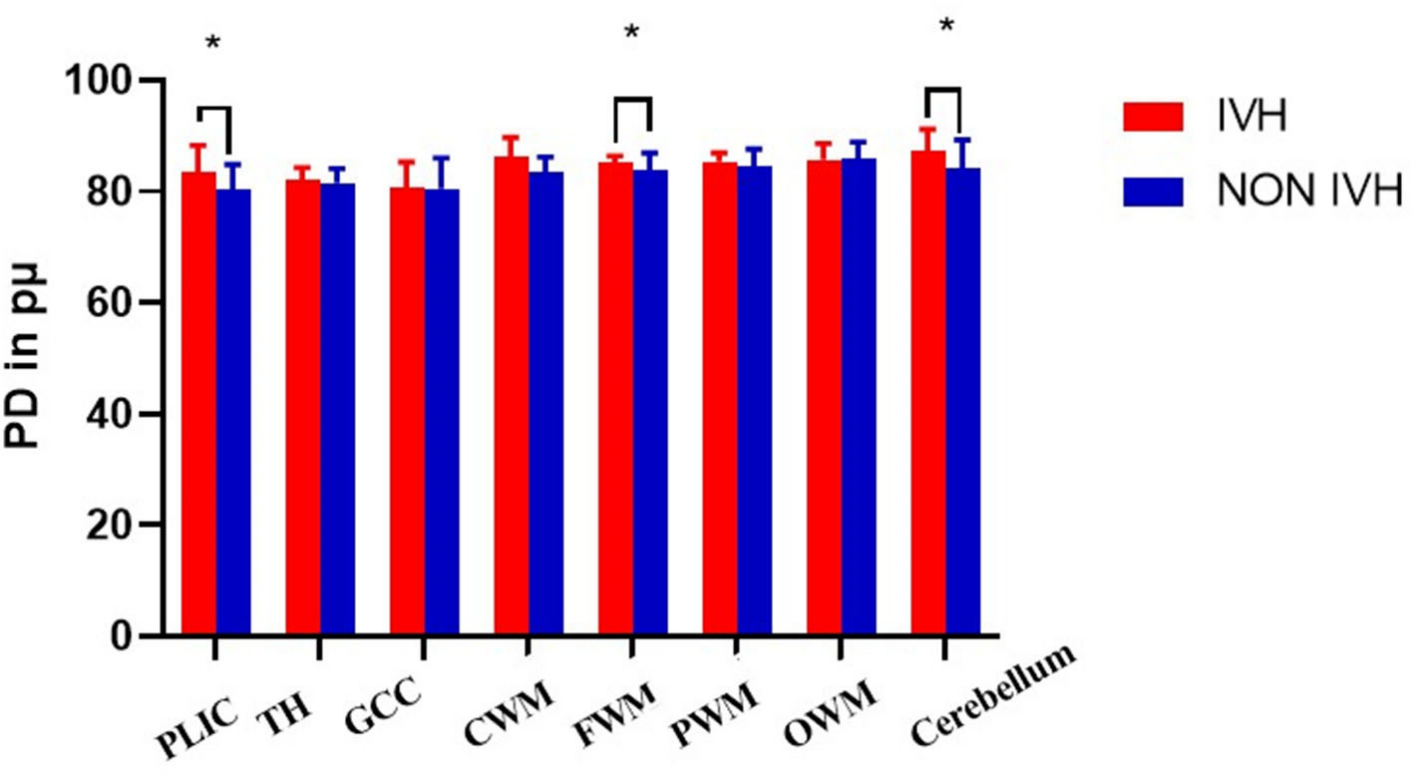
Differences in PD values between IVH and NON IVH groups. The IVH group is displayed in red and the NON IVH group in blue. PLIC, posterior limbs of the internal capsule; TH, thalamus; GCC, genu of the corpus callosum; CWM, Central white matter; FWM, frontal white matter; PWM, parietal white matter; OWM, occipital white matter. *p < 0.05, **p < 0.001.

As shown in Figure 2 and Figure 3, there was a relative difference between the regional relaxation time of the IVH and the NON IVH groups. The relaxation time was the shortest in TH (respectively, T1 = 2,011.67 ± 186.601, T2 = 231.93 ± 36.63; T1 = 1,795.46 ± 174.27, T2 = 193.40 ± 28.06) and PLIC (respectively, T1 = 1,879.80 ± 289.91, T2 = 216.40 ± 55.90; T1 = 1,786.26 ± 219.19, T2 = 165.69 ± 26.87). The parts with the longest relaxation time between the two groups were FWM (respectively, T1 = 2,011.67 ± 186.601, T2 = 231.93 ± 36.63; T1 = 1,795.46 ± 174.27, T2 = 193.40 ± 28.06) and OWM (respectively, T1 = 1,879.80 ± 289.91, T2 = 216.40 ± 55.90; T1 = 1,786.26 ± 219.19, T2 = 165.69 ± 26.87). The biggest PD value between the two groups was in the cerebellum (PD = 87.36 ± 3.86 and 84.17 ± 5.12, respectively), as shown in Figure 4.

### Tissue Volumes and Volume Fractions in IVH and NON IVH Groups

The brain tissue segmentation results of the two groups are shown in Table 3. We found that the brain tissue volume segmentation results of the IVH group were different from the NON IVH group. There were significant differences in the GM, CSF, BPV, and ICV between the two groups (all *p* < 0.001, *t* = −5.232 to 4.596). Second, the differences also existed in the NON volume (*p* = 0.010, *t* = 2.812). However, although the myelin volume in the IVH group was lower than the control group, it showed no difference (*p* = 0.166, *t* = −1.407).

**Table 3:** Volume comparison between the two groups.

	**IVH group (*n* = 15)**	**NON IVH group (*n* = 35)**	***P-*value**	***t*-value**
GM mL	260.56 ± 130.68	438.17 ± 65.15	0.000^*^	−5.004
CSF mL	119.43 ± 49.59	58.97 ± 17.80	0.000^*^	4.596
NoN mL	63.19 ± 52.52	19.30 ± 45.74	0.010^*^	2.812
Myc ml	1.22 ± 1.23	1.67 ± 0.97	0.166	−1.407
BPV ml	339.81 ± 93.7	473.36 ± 47.76	0.000^*^	−5.232
ICV ml	456.33 ± 61.07	532.48 ± 45.84	0.000^*^	−4.670

*Segmentation results for 50 subjects. GM, gray matter; CSF, cerebrospinal fluid; Myc, correlates with myelin; BPV, brain parenchymal volume; ICV, Intracranial Volume*.

* *p < 0.05 is defined as the significance level*.

**Figure 5:**
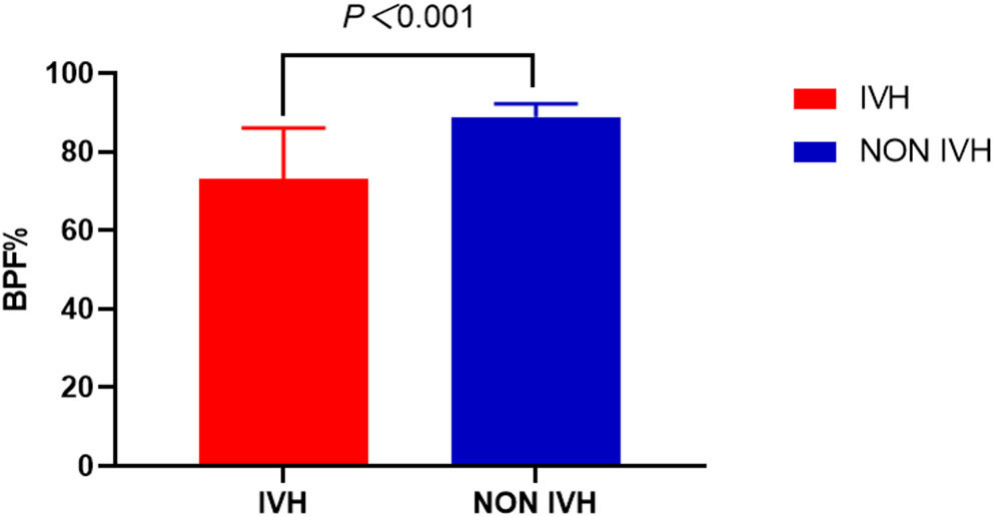
Comparison of brain parenchymal fraction between IVH and NON IVH groups. The IVH group is displayed in red and the NON IVH group in blue. *p < 0.001 is defined as the significance level.

We can also observe a similar trend in the BPF indicator. The BPF in the IVH group was significantly lower, compared with the NON IVH group (73.06 ± 13.01% and 88.87 ± 3.37%, respectively, *p* < 0.001), as shown in Figure 5.

### The Diagnostic Ability to Classify IVH Patients Based on Tissue Quantitative Values

The ROC curve and AUC were used to assess whether IVH and NON IVH could be distinguished based on local tissue relaxation time alone. The differential diagnosis ability of T1, T2, and PD measurement was found to be excellent in PLIC, CWM, and cerebellum (AUC 0.700–0.837, *p* < 0.05). Acceptable for the T1, T2, and PD measurements in PWM (AUC 0.677–0.690, all *p* < 0.05), it was insufficient for T1, T2, and PD measurements in OWM (AUC 0.566, 0.667, and 0.527, respectively). This was evaluated by the AUC of ROC curves (Table 4).

**Table 4:** The diagnostic ability of AUC for IVH disease.

	**AUC**	**95% CI**	***p*-value**
T1 PLIC	0.837	0.721–0.953	0.000^*^
T1 TH	0.752	0.589–0.916	0.005^*^
T1 GCC	0.739	0.573–0.905	0.008^*^
T1 CWM	0.731	0.555–0.908	0.010^*^
T1 FWM	0.796	0.648–0.944	0.001^*^
T1 PWM	0.690	0.527–0.852	0.035^*^
T1 OWM	0.566	0.378–0.753	0.456
T1 Cerebellum	0.813	0.670–0.957	0.000^*^
T2 PLIC	0.749	0.587–0.911	0.006^*^
T2 TH	0.589	0.419–0.758	0.325
T2 GCC	0.660	0.487–0.833	0.075
T2 CWM	0.787	0.617–0.956	0.001^*^
T2 FWM	0.820	0.684–0.956	0.300
T2 PWM	0.684	0.513–0.855	0.041^*^
T2 OWM	0.667	0.496–0.837	0.064
T2 Cerebellum	0.779	0.628–0.930	0.002^*^
PD PLIC	0.734	0.572–0.987	0.009^*^
PD TH	0.547	0.372–0.721	0.604
PD GCC	0.504	0.329–0.679	0.966
PD CWM	0.774	0.616–0.933	0.002^*^
PD FWM	0.643	0.493–0.793	0.112
PD PWM	0.677	0.525–0.829	0.049^*^
PD OWM	0.527	0.235–0.619	0.415
PD Cerebellum	0.700	0.540–0.840	0.035^*^

*Area under the ROC curve (AUC) analysis to distinguish IVH premature infants from*.

*NON IVH premature infants, based on T1 and T2 relaxation time and PD. CI, Confidence interval*.

* *p < 0.05 is defined as the significance level*.

## Discussion

Synthetic MRI can quantitatively evaluate the brain development of premature infants with intraventricular hemorrhage. In our research, the relaxation measurement showed that the T1, T2, and PD of preterm infants in the IVH group were prolonged. In addition, this paper proposed a novel method for automatic segmentation and volume estimation of whole brain tissue. Our results showed that the brain GM, CSF, BPV, and ICV of premature infants in the IVH group were significantly lower compared with the NON IVH group. The BPF results showed that, in the IVH group, BPV accounted for a small proportion of ICV, suggesting that the development was lagging behind. Relaxation measurement had a good diagnostic value for IVH premature infants, using ROC curve analysis. The research in this article shows that the quantitative value and brain volume could be obtained by quantitative synthetic MRI, which had certain value for brain development assessment and diagnosis of IVH in premature infants.

Our results revealed that the T1 and T2 relaxation times of the IVH group were significantly delayed, especially in the posterior limbs of the internal capsule, genu of the corpus callosum, frontal white matter, and central white matter. It has been reported that measuring T1 and T2 relaxation time is particularly suitable for the study of neonatal brain maturity because they reflected the changes in volumetric water content, compartmentalization, and macromolecular microstructures associated with early brain maturation and myelination (6, 7). Various microstructure changes affect T1 and T2 values (8), and the relaxation time decreases with age (9). IVH in premature infants led to the destruction and loss of glial precursor cells, and impaired cell differentiation, migration, and axon growth (10). It was consistent with our research results. IVH in premature infants affected the changes in brain water content, myelin, iron, and other components, which showed that the T1, T2, and PD values of PLIC, GCC, and other parts were prolonged. However, it was worth noting that the only significant harmful manifestations of mild GMH-IVH involved the development of the cortex and deep gray matter, which were found in MRI studies (11). This view was consistent with the different results shown by PLIC and TH in this study. At the same time, our research results demonstrated that there were also significant differences in the relaxation time of the cerebellum between the two groups. Some studies believed that hemosiderin caused by IVH leaked into the CSF, which affected the cerebellar development of premature infants (12). Therefore, we speculated that the development of the cerebellum in children with IVH might affect their exercise and balance ability.

Generally speaking, the order of white matter myelination is bottom–up, back-to-front, and center-to-around (13). The PLIC is the first region where the telencephalon begins to myelinate. At the same time, as the brain continues to develop, myelin sheath increases and water decreases. This leads to a decrease in T1 and T2 values. Therefore, this study shows that for the partially myelinated PLIC, TH exhibited the shortest relaxation time. However, FWM and OWM, which developed later had the longest relaxation time of T1 and T2. The above results indicated that synthetic MRI had a certain value in discovering the changes in the microstructure of the brain and quantitatively assessing the brain damage in children with IVH.

Brain MRI segmentation and brain volume estimation are of great significance in many neurological applications (14). Our results showed that the GM, CSF, brain parenchyma, and total brain volume of IVH children were significantly lower than those of the NON IVH group. Studies showed that after the 24th week of pregnancy, neurons completed their migration, but the germinal matrix still provided glial precursors to form brain oligodendrocytes and astrocytes. In the late stage of glial formation, astrocytes migrated to the upper cortex, which is essential for neuronal survival and normal cerebral cortex development (10, 15). The destruction of IVH may lead to a decrease in the number of GABA neurons, which explained the decrease in cortical volume. Thus, the GM volume was significantly reduced in IVH children. After the occurrence of IVH, germ cell proliferation stopped in the ganglion bulge; meanwhile, thrombin, plasminase, and toxic neurotransmitters increased (16). The extracellular hemoglobin rapidly oxidized from a ferrous state to a highly reactive iron state. Proliferation, differentiation, and axon growth of germ cells were then prevented by activating cytotoxic, oxidative, and inflammatory pathways (17, 18). Another study showed that their cerebral cortex was composed of promyelin oligodendrocytes and oligodendrocyte precursor cells, for premature babies <32 weeks after birth. However, premyelinated oligodendrocytes were more susceptible to excitotoxicity and oxidative stress than mature myelin oligodendrocytes (19, 20). These views were in line with the results of our study. Children with IVH had lower BPV and

ICV compared with the control group. Our results showed no significant difference in myelin sheath volume compared with the control group. We speculated that the reason might be that the germinal matrix was the main source of oligodendrocyte progenitor proliferation. Moreover, oligodendrocyte progenitor cells migrated to the white matter, differentiated, and produced cerebral myelin in the third trimester. IVH, however, damaged the germinal matrix, thus, resulting in the loss of myelin-producing cells. The final manifestations were impaired myelin development and neurodevelopment. At the same time, the premature infants in the control group had a smaller corrected gestational age, and only partially myelinated or unmyelinated, so the difference in myelin volume was not significant.

The BPF is calculated as the ratio of BPV and ICV. The brain parenchyma of IVH patients occupied a smaller volume in the skull. This indicated that the brain development of children with IVH was lagging behind. In the long run, it will affect children's intellectual development and abnormal behavior, language disorders, etc. (21). Therefore, the quantitative estimation of brain tissue volume using MRI was beneficial to the evaluation of brain development and disease diagnosis in IVH children.

In the central nervous system, quantitative biomarkers are increasingly needed to identify early brain injury or other neurological diseases. Synthetic MRI relaxometry of T1, T2, and PD can be used to quantitatively detect the differences in brain microstructure (22, 23). Our ROC curve analysis results show that T1, T2, and PD indicators could better diagnose IVH in premature infants, especially in the PLIC, CWM, and cerebellum. We speculated that the PLIC and CWM were myelinated earlier, resulting in higher metabolic rate and oxygen consumption. Oxidative stress and cytotoxic edema occurred during intracranial hemorrhage, which first affected these areas. IVH caused a leakage of hemosiderin into the CSF. They were deposited on the surface of the cerebellum and disrupted its structure. Therefore, our results show that the relaxation time of the cerebellar in IVH children could also be a valid indicator for the diagnosis. Synthetic MRI had excellent characteristics for diagnosing neonatal brain damage, while conventional MRI had limitations in predicting the prognosis of extremely premature infants (24). Therefore, we believed that synthetic MRI might provide early prognostic biomarkers for neurodevelopmental disorders and had certain significance for the IVH diagnosis.

The following limitations merit consideration. First of all, the sample size included in the case group was small. This requires subsequent collection of more IVH patients to increase the sample size. Second, the manually drawn ROI was subjective. Afterward, it is expected to automatically delineate the ROIs. In addition, studies reported that the artifacts of T2 FLAIR displayed in synthetic MRI were more common, but these artifacts were easy to identify and did not affect the diagnosis (25). Therefore, it is recommended to combine multiple images to comprehensively diagnose IVH disease.

## Conclusion

Synthetic MRI is very sensitive in detecting the changes of brain microstructure in children with IVH. It can quantitatively determine the relaxation time and the changes of brain volume. We observed significant prolongation of local T1, T2, and PD in IVH premature infants. At the same time, the GM volume, total brain volume, and brain parenchymal fraction were also significantly reduced. Quantitative parameters of quantitative synthetic MRI were helpful to distinguish the brain tissue of premature infants with IVH from that of normal premature infants. Synthetic MRI may provide early biomarkers for neurodevelopmental disorders.

## Data Availability Statement

The original contributions presented in the study are included in the article/supplementary material, further inquiries can be directed to the corresponding author/s.

## Ethics Statement

The studies involving human participants were reviewed and approved by Ethics Committee of the Third Affiliated Hospital of Zhengzhou University. Written informed consent to participate in this study was provided by the participants' legal guardian/next of kin.

## Author Contributions

CZ: manuscript editing. MC: statistical analysis. XinZ: study design and work supervising. XiaZ: study concepts, design, and funding. KW: language modification. All authors contributed to the article and approved the submitted version.

## Funding

This research was funded by The National Natural Science Foundation of China, Grant No. 81870983. At the same time, it was also funded by Scientific and technological key project of Henan Province, Grant No. 212102310742.

## Conflict of Interest

KW was employed by company GE Healthcare. The remaining authors declare that the research was conducted in the absence of any commercial or financial relationships that could be construed as a potential conflict of interest.

## Publisher's Note

All claims expressed in this article are solely those of the authors and do not necessarily represent those of their affiliated organizations, or those of the publisher, the editors and the reviewers. Any product that may be evaluated in this article, or claim that may be made by its manufacturer, is not guaranteed or endorsed by the publisher.
